# Influence of glycated hemoglobin on thromboinflammation in acute ischemic stroke: a retrospective, propensity score matching study

**DOI:** 10.3389/fendo.2025.1542549

**Published:** 2025-07-28

**Authors:** Liuding Wang, Yifan Chen, Longtao Liu, Min Jia, Yunfan Zhang, Ze Chang, Zhiyi Gong, Jian Lyu, Xiao Liang, Yunling Zhang

**Affiliations:** ^1^ Xiyuan Hospital, China Academy of Chinese Medical Sciences, Beijing, China; ^2^ Departmalet of Neurology, Xiyuan Hospital, China Academy of Chinese Medical Sciences, Beijing, China; ^3^ Departmalet of Cardiology, Xiyuan Hospital, China Academy of Chinese Medical Sciences, Beijing, China; ^4^ Guang’anmen Hospital, China Academy of Chinese Medical Sciences, Beijing, China; ^5^ Medical Ethics Committee, Xiyuan Hospital, China Academy of Chinese Medical Sciences, Beijing, China; ^6^ Graduate School, Beijing University of Chinese Medicine, Beijing, China; ^7^ Shandong University of Traditional Chinese Medicine, Jinan, China; ^8^ National Medical Products Administration (NMPA) Key Laboratory for Clinical Research and Evaluation of Traditional Chinese Medicine, Xiyuan Hospital, China Academy of Chinese Medical Sciences, Beijing, China; ^9^ National Clinical Research Center for Chinese Medicine Cardiology, Xiyuan Hospital, China Academy of Chinese Medical Sciences, Beijing, China

**Keywords:** diabetes, cerebral infarction, coagulation dysfunction, immune thrombosis, real world study

## Abstract

**Background:**

Hyperglycemia is acknowledged as a pivotal factor associated with poor prognosis in acute ischemic stroke (AIS). The intricate interplay among hyperglycemia, thrombosis, and inflammation has garnered significant attention. Therefore, we aimed to investigate the association between hemoglobin A1c (HbA1c) and risk of thrombosis, and the role of inflammation, in patients with AIS.

**Methods:**

A total of 1,291 patients with AIS were identified from Xiyuan Hospital, China Academy of Chinese Medical Sciences. A propensity score matching was used to address baseline imbalances. AIS patients were divided into a high HbA1c group (n = 419) and a control group (n = 656) based on whether their initial HbA1c levels upon admission were above or below 6.5%. Thrombosis was assessed using coagulation parameters. Inflammation was reflected by markers such as the neutrophil-to-lymphocyte ratio (NLR), systemic immune-inflammation index (SII), and systemic inflammatory response index (SIRI). Chi-square test, independent sample t-test, Mann-Whitney U test, and logistic regression were used for correlation analysis.

**Results:**

In AIS patients, HbA1c levels > 6.5% were significantly associated with abnormal coagulation function and elevated inflammatory response. Among AIS patients with elevated HbA1c, high fibrinogen levels were significantly correlated with increased inflammatory markers such as SII and SIRI. Furthermore, HbA1c > 6.5% was identified as an independent predictor for hypercoagulability in AIS patients (*OR* = 1.74, 95% CI 1.17 − 2.60, *P* = 0.006).

**Conclusions:**

Elevated HbA1c levels were associated with severe hypercoagulability and heightened inflammatory responses following AIS onset. Elevated HbA1c levels may contribute to poorer outcomes, likely due to the thromboinflammation.

## 1 Introduction

Hyperglycemia is a risk factor for adverse prognosis in patients with acute ischemic stroke (AIS) (1). Compared to acute blood glucose levels or diabetes history, glycosylated hemoglobin A1c (HbA1c) levels might be more appropriate for predicting symptomatic hemorrhage and early neurological deterioration following recanalization therapy in AIS (2, 3). HbA1c is a widely recognized biomarker for monitoring long-term blood glucose levels, with elevated levels indicating clear vascular damage over the past three months. Previous studies have found that hyperglycemia may worsen AIS by inducing inflammation and endothelial dysfunction, but the biological mechanisms linking hyperglycemia, inflammation, and vascular damage remain unclear (4). A population-based study indicated that in 5778 patients, HbA1c levels were positively correlated with the activity levels of coagulation factors VIII, IX, and XI, as well as fibrinogen levels (5). In recent years, the interaction between coagulation abnormalities and inflammation, leading to thromboinflammation, has become a research focus in the pathophysiological mechanisms of AIS (6). Recent research indicates that the improvement in long-term functional outcomes in hyperglycemic mice following stroke might be related to the reduction of thromboinflammation (7). Considering the potential effects of glucose metabolism on coagulation factors and the key role of thromboinflammation in AIS progression, this study analyzed the impact of HbA1c levels on coagulation function and inflammatory markers in patients with AIS, explored the predictive value of HbA1c levels for coagulation abnormalities, and discussed how hyperglycemia might influence AIS prognosis by worsening thromboinflammation.

## 2 Materials and methods

### 2.1 Study design and patients

This is a retrospective cohort study, and the study protocol was approved by the Medical Ethics Committee of Xiyuan Hospital, China Academy of Chinese Medical Sciences (approval number: 2020XLA054-4). The patients were admitted to Xiyuan Hospital, China Academy of Chinese Medical Sciences, for AIS treatment between January 2015 and December 2019. The diagnosis of AIS was based on the “Chinese Acute Ischemic Stroke Diagnosis and Treatment Guidelines 2018” (8): (1) acute onset; (2) focal neurological deficits, with some cases showing global neurological deficits; (3) imaging revealing the responsible lesion or symptoms/signs lasting for over 24 hours; (4) exclusion of non-vascular causes; (5) brain CT/MRI to rule out intracranial hemorrhage.

Inclusion criteria: (1) age ≥ 60 years, with no gender restrictions; (2) meets the diagnostic criteria for AIS and is in the acute phase upon admission; (3) complete documentation of the medical record. Exclusion criteria: (1) presence of active infections upon admission, such as bacterial pneumonia, aspiration pneumonia, chronic obstructive pulmonary disease with acute lower respiratory tract infections, bronchiectasis with infection, urinary tract infections, etc.; (2) concomitant severe hematological diseases, such as aplastic anemia, thrombocytopenic purpura, etc.; (3) concomitant autoimmune diseases, such as rheumatoid arthritis, systemic lupus erythematosus, rheumatoid arthritis, and rheumatic heart disease.

### 2.2 Research methods

Demographic characteristics of patients (age, gender), chief complaints, admission and discharge diagnoses, as well as the first laboratory test results upon admission, including complete blood count, blood biochemistry, HbA1c, and the four coagulation parameters [prothrombin time (PT), activated partial thromboplastin time (APTT), thrombin time (TT), fibrinogen (Fbg)], were retrieved and documented from the inpatient electronic medical record system. Based on the HbA1c level detected after admission, indicating whether the patient has been exposed to hyperglycemia in the past three months, AIS patients were categorized into the study group (HbA1c > 6.5%) and the control group (HbA1c ≤ 6.5%). According to current international guidelines, an HbA1c level > 6.5% indicates elevated HbA1c levels, suggesting the presence of diabetes and poor glycemic control.

Coagulation function parameters include PT, APTT, TT, and Fbg. Inflammatory markers include absolute neutrophil count (ANC), absolute lymphocyte count (ALC), absolute monocyte count (AMC), neutrophil-to-lymphocyte ratio (NLR), systemic immune-inflammation index (SII), and systemic inflammation response index (SIRI). Reference range values: PT 10.00~14.00 sec, APTT 20.00~40.00 sec, TT 13.00~21.00 sec, Fbg 1.70~4.05 g/L, ANC 1.80~6.30×10^9^/L, ALC 1.10~3.20×10^9^/L, AMC 0.10~0.60×10^9^/L. The formula for calculating NLR is NLR = ANC/ALC. The formula for calculating SII is SII = absolute platelet count×ANC/ALC. The formula for calculating SIRI is SIRI = ANC×AMC/ALC. Several predictive model studies (9–12) have shown that elevated values of NLR, SII, and SIRI are independent risk factors for poor prognosis in AIS, with the optimal cutoff values possibly being 4.20, 545.14×10^9^/L, and 1.298×10^9^/L, respectively.

### 2.3 Statistical analysis

Statistical analysis was performed using IBM SPSS 26.0 software. A 1:2 propensity score matching (PSM) was used to address baseline imbalances. We matched participants on age, sex, comorbidities, anticoagulant and antiplatelet therapies using a caliper of 0.02. Categorical variables were described by frequency and percentage, and group comparisons were performed using the χ² test; continuous variables with a normal distribution were described using “mean ± standard deviation,” with comparisons between groups conducted using the independent t-test and ANOVA for multiple groups; non-normally distributed continuous variables were described using “median (interquartile range)” and compared between two groups using the Mann-Whitney U test, and between multiple groups using the Kruskal-Wallis test. Statistical significance was defined as *P* < 0.05. Subgroup analysis of the effect of HbA1c was performed based on comorbidities. A correlation analysis of coagulation and inflammatory changes was conducted in AIS patients with high HbA1c levels. Binary logistic regression was performed to analyze the risk factors for coagulation dysfunction in AIS. Collinearity diagnostics were performed to examine interactions among variables. The predictive performance was assessed by constructing receiver operating characteristic (ROC) curves and computing the corresponding area under the curve (AUC).

## 3 Results

### 3.1 Baseline characteristics

A total of 1,291 patients with AIS were included in this study. Laboratory results indicated that 473 patients (36.64%) had HbA1c > 6.5%, of which 203 had a history of diabetes; 818 patients (63.36%) had HbA1c ≤ 6.5%, with 361 having a history of diabetes. **Table 1** summarizes the differences in sociodemographic characteristics, comorbidities, medication use, and selected biochemical results between the two groups. No statistically significant differences were found between the two groups in terms of age, gender, history of hypertension, history of diabetes, history of dyslipidemia, and current smoking (*P* > 0.05). Patients with elevated HbA1c had significantly lower atrial fibrillation rates (*P* < 0.05) but higher antiplatelet and hypoglycemic medication use (*P* < 0.05) compared to controls.

**Table 1:** Comparison of baseline characteristics between the study group and control group.

Baseline characteristics	HbA1c > 6.5% (n = 473)	HbA1c ≤ 6.5% (n = 818)	*P* value
Demographic characteristics			
Age, y; media (Q1, Q3)	74 (66, 80)	74 (66, 81)	0.199
Male; n (%)	299 (63.21)	476 (58.19)	0.076
Comorbidities			
Diabetes; n (%)	203 (42.92)	361 (44.13)	0.672
Hypertension; n (%)	383 (80.97)	668 (81.66)	0.759
Dyslipidemia; n (%)	224 (47.36)	362 (44.25)	0.281
Atrial fibrillation; n (%)	28 (5.92)	92 (11.25)	0.001
Renal insufficiency; n (%)	43 (9.09)	72 (8.80)	0.861
Heart failure; n (%)	17 (3.59)	28 (3.42)	0.872
Regular medication			
Warfarin; n (%)	4 (0.85)	5 (0.61)	0.626
Rivaroxaban; n (%)	1 (0.21)	3 (0.37)	0.629
Clopidogrel; n (%)	27 (5.71)	24 (2.93)	0.014
Aspirin; n (%)	76 (16.07)	95 (11.61)	0.023
Hypoglycemic therapy; n (%)	298 (63.00)	104 (12.71)	0.001
Alcohol consumption and smoking status			
Current drinker; n (%)	94 (19.87)	180 (22.00)	0.367
Current smoker; n (%)	95 (20.08)	191 (23.35)	0.173

### 3.2 Propensity score matching

To adjust for confounding factors, we performed PSM based on age, sex, comorbidities, medication use, and smoking/alcohol habits. After matching, the study group included 419 cases and the control group included 656 cases (**Table 2**). The between-group statistical differences in clopidogrel use (*P* = 0.203) and aspirin use (*P* = 0.448) were eliminated, and the *P*-values for sex and smoking also improved significantly. Although the difference in hypoglycemia therapy remained significant, we considered that this factor was closely associated with HbA1c > 6.5% and thus did not require further balancing. Overall, the confounding factors between the groups were effectively controlled after propensity score matching.

**Table 2:** Comparison of baseline characteristics between the two groups after propensity score matching.

Baseline characteristics	HbA1c > 6.5% (n = 419)	HbA1c ≤ 6.5% (n = 656)	*P* value
Demographic characteristics			
Age, y; media (Q1, Q3)	74 (66, 80)	73 (65, 80)	0.993
Male; n (%)	259 (61.81)	404 (61.59)	0.940
Comorbidities			
Diabetes; n (%)	181 (43.20)	289 (44.05)	0.782
Hypertension; n (%)	335 (79.95)	542 (82.62)	0.271
Dyslipidemia; n (%)	188 (44.87)	298 (45.43)	0.858
Atrial fibrillation; n (%)	26 (6.21)	69 (10.52)	0.015
Renal insufficiency; n (%)	39 (9.31)	56 (8.54)	0.664
Heart failure; n (%)	14 (3.34)	24 (3.66)	0.784
Regular medication			
Warfarin; n (%)	3 (0.72)	4 (0.61)	0.833
Rivaroxaban; n (%)	0 (0.00)	0 (0.00)	–
Clopidogrel; n (%)	11 (2.63)	10 (1.52)	0.203
Aspirin; n (%)	51 (12.17)	70 (10.67)	0.448
Hypoglycemic therapy; n (%)	258 (61.58)	84 (12.80)	0.001
Alcohol consumption and smoking status			
Current drinker; n (%)	81 (19.33)	139 (21.19)	0.462
Current smoker; n (%)	84 (20.05)	137 (20.88)	0.741

### 3.3 Impact of HbA1c levels on coagulation function in patients with AIS

Compared to the control group, the high HbA1c group had significantly lower PT and APTT, and significantly higher Fbg levels, with differences being statistically significant (*P* < 0.05, **Table 3**). No statistically significant difference was found in TT between the two groups. The proportion of patients in the high HbA1c group with PT < 10.00 sec was significantly higher than in the control group (5.49% *vs*. 3.05%, *P* = 0.047), and the proportion of patients with Fbg > 4.05 g/L was significantly higher in the high HbA1c group compared to the control group (13.6% *vs*. 8.23%, *P* = 0.005). No statistically significant difference was found between the groups regarding the proportion of patients with APTT < 20.00 sec. No patients in either group had TT below the lower reference range.

**Table 3:** Impact of HbA1c > 6.5% on coagulation function parameters.

Coagulation function	HbA1c > 6.5% (n = 419)	HbA1c ≤ 6.5% (n = 656)	*P* value
Continuous variables			
PT, sec; media (Q1, Q3)	11.10 (10.60, 11.60)	11.30 (10.80, 11.90)	< 0.001
APTT, sec; media (Q1, Q3)	27.00 (25.00, 29.20)	28.30 (26.00, 30.88)	< 0.001
TT, sec; media (Q1, Q3)	18.80 (18.10, 19.50)	18.80 (18.03, 19.50)	0.602
Fbg, g/L; media (Q1, Q3)	2.92 (2.44, 3.51)	2.74 (2.27, 3.32)	0.001
Categorical variables			
PT < 10.00 sec; n (%)	23 (5.49)	20 (3.05)	0.047
APTT < 20.00 sec; n (%)	3 (0.72)	2 (0.3)	0.334
Fbg > 4.05 g/L; n (%)	57 (13.6)	54 (8.23)	0.005

APTT, activated partial thromboplastin time; Fbg, fibrinogen; PT, prothrombin time; TT, thrombin time.

### 3.4 Impact of HbA1c levels on inflammatory response in patients with AIS

Compared to the control group, the high HbA1c group had significantly elevated ANC, ALC, and SII, with statistically significant differences (*P* < 0.05, **Table 4**). No significant difference was observed in AMC, APC, NLR, and SIRI between the two groups.

**Table 4:** Impact of HbA1c > 6.5% on inflammatory response parameters.

Inflammatory response	HbA1c > 6.5% (n = 419)	HbA1c ≤ 6.5% (n = 656)	*P* value
Continuous variables			
ANC, 10^9^/L; media (Q1, Q3)	4.40 (3.50, 5.57)	3.95 (3.11, 5.08)	< 0.001
ALC, 10^9^/L; media (Q1, Q3)	1.66 (1.27, 2.10)	1.54 (1.17, 1.97)	0.003
AMC, 10^9^/L; media (Q1, Q3)	0.39 (0.31, 0.51)	0.39 (0.31, 0.50)	0.859
APC, 10^9^/L; media (Q1, Q3)	222.00 (185.00, 266.00)	216.50 (183.25, 260.75)	0.2
NLR; media (Q1, Q3)	2.65 (1.95, 3.85)	2.44 (1.83, 3.55)	0.062
SII, 10^9^/L; media (Q1, Q3)	594.50 (414.92, 879.29)	527.50 (374.26, 822.92)	0.024
SIRI, 10^9^/L; media (Q1, Q3)	1.03 (0.65, 1.78)	0.94 (0.61, 1.52)	0.142
Categorical variables			
NLR > 4.20; n (%)	93 (22.5)	123 (18.75)	0.169
SII > 545.14×10^9^/L; n (%)	241 (57.52)	314 (47.87)	0.002
SIRI > 1.298×10^9^/L; n (%)	161 (38.42)	202 (30.79)	0.01

ANC, absolute neutrophil count; ALC, absolute lymphocyte count; AMC, absolute monocyte count; APC, absolute platelet count; NLR, neutrophil-to-lymphocyte ratio; SII, systemic immune-inflammation index; SIRI, systemic inflammation response index.

The proportion of patients in the high HbA1c group with SII > 545.14×10^9^/L was significantly higher than in the control group (57.52% *vs*. 47.87%, *P* = 0.002), and the proportion of patients with SIRI > 1.298×10^9^/L was also significantly higher in the high HbA1c group compared to the control group (38.42% *vs*. 30.79%, *P* = 0.01). No statistically significant difference was observed in the proportion of patients with NLR > 4.2 between the two groups.

### 3.5 Subgroup analysis of HbA1c-associated coagulation dysfunction in patients with AIS

Elevated HbA1c showed selective associations with higher fibrinogen (Fbg > 4.05 g/L) (**Table 5**). Significant positive correlations emerged in patients with hypertension, dyslipidemia, or renal insufficiency (*P* < 0.05), but not in those without these conditions. The association was stronger in heart failure patients than in those without heart failure. Notably, while non-diabetics demonstrated a robust HbA1c-Fbg relationship, this association disappeared in established diabetes.

**Table 5:** Subgroup analysis of coagulation function in AIS patients with HbA1c > 6.5% versus those with HbA1c ≤ 6.5%.

Subgroups	HbA1c > 6.5%	HbA1c ≤ 6.5%	*P* value
	Fbg > 4.05, n (%)		
Hypertension			
Yes (n = 877)	48 (14.33)	46 (8.49)	0.007
No (n = 198)	9 (10.71)	8 (7.02)	0.36
Dyslipidemia			
Yes (n = 486)	31 (16.49)	28 (9.4)	0.02
No (n = 589)	26 (11.26)	26 (7.26)	0.096
Renal insufficiency			
Yes (n = 95)	16 (41.03)	7 (12.5)	0.001
No (n = 980)	41 (10.79)	47 (7.83)	0.115
Heart failure			
Yes (n = 38)	5 (35.71)	2 (8.33)	0.038
No (n = 1037)	52 (12.84)	52 (8.23)	0.016
Diabetes			
Yes (n = 470)	20 (11.05)	27 (9.34)	0.549
No (n = 605)	37 (15.55)	27 (7.36)	0.001

Fbg, fibrinogen.

### 3.6 Correlation analysis between coagulation function and inflammatory response

Using the upper reference value for Fbg (4.05 g/L) as the cutoff, the AIS patients with HbA1c > 6.5% were divided into two groups: coagulation dysfunction (65 cases) and normal coagulation function (408 cases). In the coagulation dysfunction group, NLR, SII, and SIRI were all significantly higher, with statistically significant differences (*P* < 0.05, **Table 6**). For AIS patients with HbA1c ≤ 6.5%, coagulation dysfunction was also significantly correlated with high levels of inflammation (**Table 6**).

**Table 6:** Correlation between coagulation function and inflammation in AIS patients.

Inflammatory response	Fbg > 4.05	Fbg ≤ 4.05	*P* value
HbA1c > 6.5%			
NLR; media (Q1, Q3)	4.65 (2.77, 6.92)	2.35 (1.77, 3.36)	< 0.001
SII, 10^9^/L; media (Q1, Q3)	1013.11 (705.18, 1569.66)	505.04 (360.73, 763.20)	< 0.001
SIRI, 10^9^/L; media (Q1, Q3)	2.22 (1.13, 4.06)	0.88 (0.60, 1.37)	< 0.001
HbA1c ≤ 6.5%			
NLR; media (Q1, Q3)	3.82 (2.60, 7.35)	2.51 (1.90, 3.55)	< 0.001
SII, 10^9^/L; media (Q1, Q3)	969.53 (584.32, 1548.19)	564.68 (386.91, 798.67)	< 0.001
SIRI, 10^9^/L; media (Q1, Q3)	1.70 (1.01, 3.64)	0.95 (0.61, 1.65)	< 0.001

NLR, neutrophil-to-lymphocyte ratio; SII, systemic immune-inflammation index; SIRI, systemic inflammation response index.

### 3.7 Risk factor analysis for coagulation dysfunction in patients with AIS

**Figure 1:**
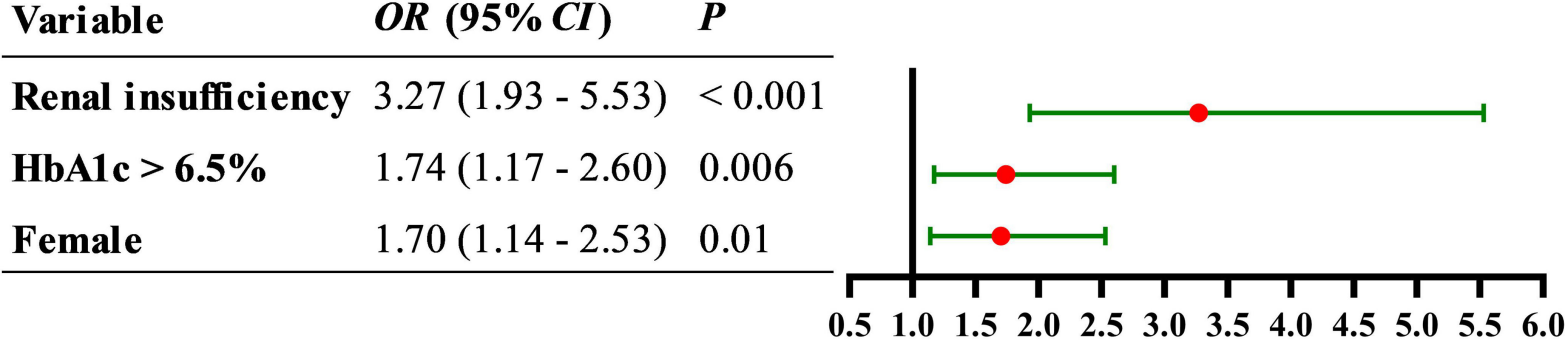
Forest plot of the association between female, elevated HbA1c, and renal insufficiency with the risk of coagulation dysfunction in patients with AIS.

**Figure 2:**
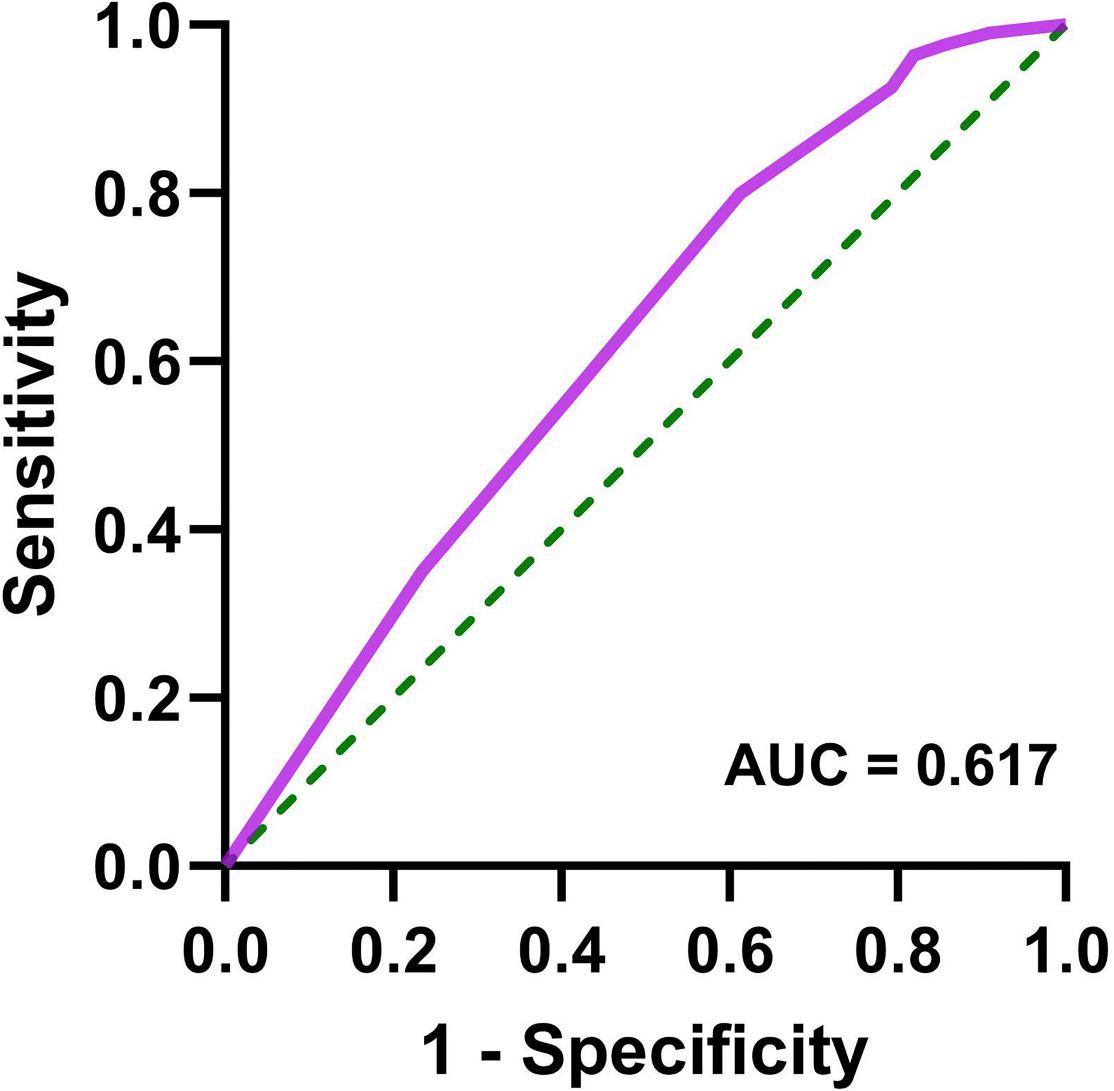
Receiver operating characteristic curve for logistic regression model (female, HbA1c > 6.5%, and renal insufficiency) in predicting coagulation dysfunction in patients with AIS.

**Figure 3:**
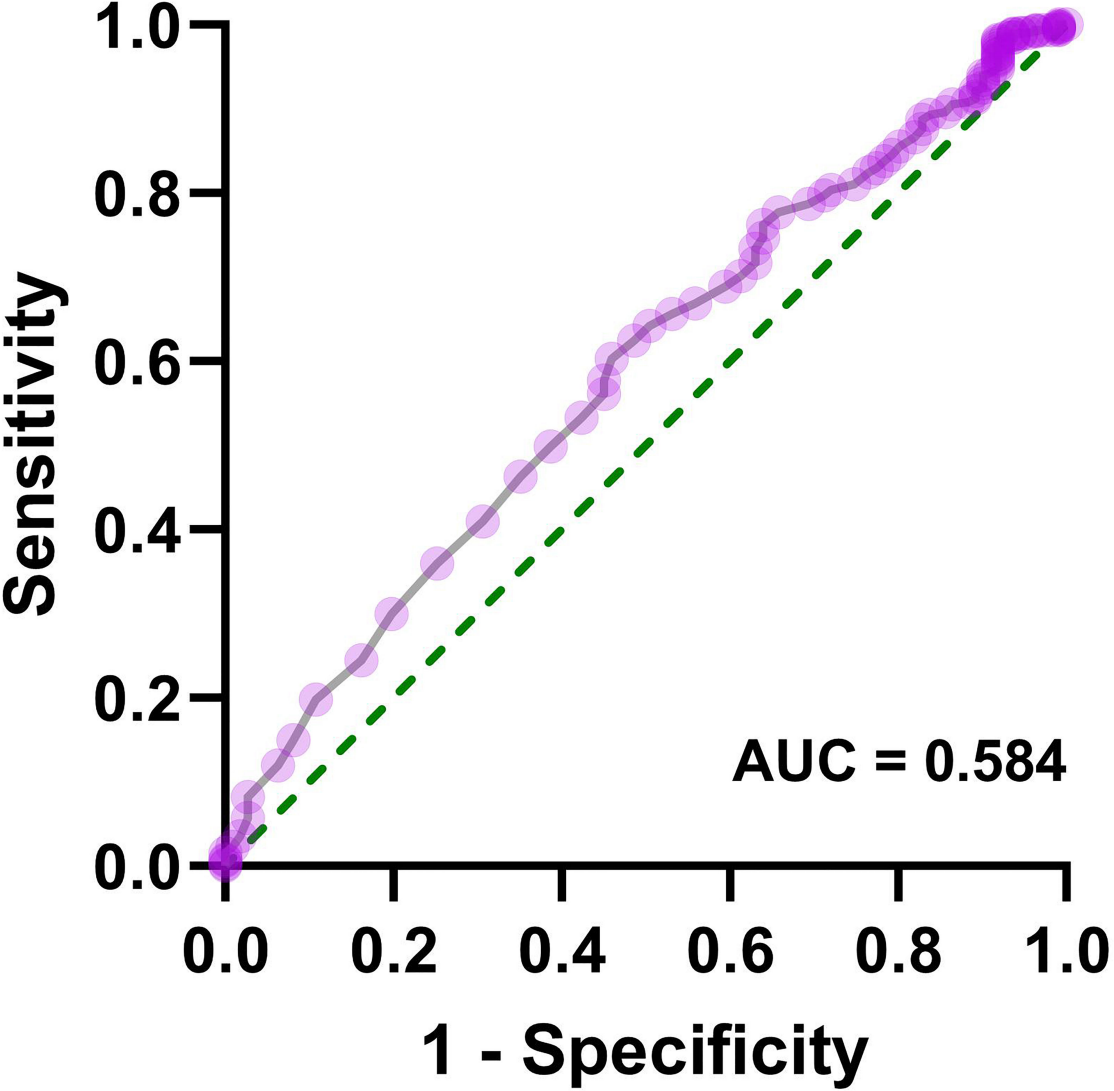
Receiver operating characteristic curve for HbA1c as a continuous variable in predicting coagulation dysfunction in patients with AIS.

Patients with AIS were divided into two groups: coagulation dysfunction group (Fbg > 4.05 g/L, n = 111) and control group (Fbg ≤ 4.05 g/L, n = 964). The proportion of patients with coagulation dysfunction was 10.33%. The ANOVA results revealed significant between-group differences in three variables: elevated HbA1c (*P* = 0.005), sex (*P* = 0.01), and renal insufficiency (*P* =0.000). Age, smoking and drinking status, medication use, and other comorbidities showed no significant group differences. A binary logistic regression analysis was performed with coagulation dysfunction as the dependent variable and HbA1c > 6.5%, sex, and renal insufficiency as independent variables. Collinearity diagnostics indicated no significant multicollinearity among these independent variables (variance inflation factor = 1). Logistic regression analysis showed that renal insufficiency (*OR* = 3.27, 95% CI 1.93 − 5.53, *P* < 0.001), HbA1c > 6.5% (*OR* = 1.74, 95% CI 1.17 − 2.60, *P* = 0.006), and female (*OR* = 1.70, 95% CI 1.14 − 2.53, *P* = 0.01) were significant risk factors for coagulation dysfunction in patients with AIS (**Figure 1**). The logistic regression model achieved an AUC of 0.617 (95% CI 0.558 − 0.676, *P* < 0.001) (**Figure 2**). ROC curve analysis of HbA1c as a continuous variable yielded an AUC of 0.584 (95% CI 0.529 − 0.639, *P* = 0.004) (**Figure 3**). The optimal cutoff value was determined as 6.45% using Youden’s index (0.144).

## 4 Discussion

This study, for the first time, highlights the significant effect of high HbA1c levels on thromboinflammation in AIS and investigates the correlation between coagulation function and inflammation response. The study indicated that HbA1c levels > 6.5% were significantly associated with coagulation dysfunction and an heightened inflammatory response during the acute phase of ischemic stroke. Regardless of HbA1c levels, hyperfibrinogenemia in AIS patients was significantly correlated with increased inflammatory markers such as NLR, SII, and SIRI. Additionally, through univariate screening and subsequent binary logistic regression analysis, we identified HbA1c > 6.5%, renal insufficiency, and female sex as independent predictive factors for coagulation dysfunction in patients with AIS. The risk of hyperfibrinogenemia in patients with HbA1c > 6.5% is 1.75 times higher than in patients with normal HbA1c levels. These findings demonstrate that poor pre-stroke glycemic control significantly exacerbates both hypercoagulability and excessive inflammatory responses. A previous prospective cohort study identified elevated HbA1c level as an independent predictor of poor clinical outcomes in large vessel occlusion patients receiving mechanical thrombectomy (2). Several clinical studies found that pre-admission or admission hyperglycemia is a key predictor of poor short- and long-term outcomes in AIS patients (13–16). This study offers a new mechanistic perspective on the biological basis of poor blood glucose control in AIS patients, particularly in terms of coagulation and inflammation. Previous studies have found that a clear history of diabetes, high blood sugar levels at admission, stress-induced hyperglycemia, and chronic hyperglycemia increase the risk of poor prognosis in acute coronary syndrome (ACS) patients, and suggest that this may be related to fibrinolysis resistance, enhanced platelet activation, and increased thrombin generation leading to thrombosis (17–20). This study found that elevated blood glucose levels in the past three months in AIS patients are also closely associated with fibrinolysis resistance. Thus, this study, along with previous research, indicates that high blood glucose levels are a significant risk factor for recurrent adverse cardiovascular events or death in ischemic cardiovascular and cerebrovascular diseases.

Previous studies have found that hyperglycemia can exacerbate blood-brain barrier damage, promote hemorrhagic transformation, and lead to early neurological deterioration in AIS patients (21, 22). The key pathological mechanisms involved may include endothelial cell ferroptosis, neuroinflammation, and mitochondrial electron transport chain dysfunction (23–26), which are closely related to signaling pathways such as P2RX7-ERK1/2, AGE-RAGE, and NLRP3. This study found that chronic hyperglycemia can cause coagulation dysfunction at admission in AIS patients, manifested by elevated fibrinogen (Fbg) levels and shortened prothrombin time (PT), suggesting that fibrinolysis resistance and hypercoagulability may be key pathological mechanisms for poor prognosis in AIS patients with high blood sugar. In individuals with chronic hyperglycemia, pathological phenomena related to fibrin formation and lysis abnormalities occur, such as increased fibrin network density, decreased fibrin clot permeability, and impaired fibrinolysis (27, 28). Compared to non-diabetic patients, diabetic patients have a higher proportion of thrombotic fibrin and lower permeability blood clots (29, 30). Studies have shown that fibrin-rich thrombi are significantly associated with prolonged reperfusion surgery time and poor prognosis in AIS patients (31). In addition to fibrin abnormalities, hyperglycemia can also increase tissue factor-induced procoagulant activity (32). Furthermore, this study found that chronic hyperglycemia is significantly correlated with increased inflammation markers such as NLR, SII, and SIRI at admission in AIS patients, and that changes in coagulation and inflammation markers are highly correlated. This suggests that thromboinflammation may be an important pathological event influencing the prognosis of AIS patients with high HbA1c levels. In recent years, researchers have increasingly focused on the complex interplay between thrombosis formation and inflammation, emphasizing the importance of thromboinflammation in the development of cardiovascular and cerebrovascular diseases (33). Previous studies have shown that elevated fibrinogen levels in COVID-19 patients are significantly associated with increases in inflammatory markers such as interleukin-6, C-reactive protein, ferritin, erythrocyte sedimentation rate, and procalcitonin (34, 35). Fibrinogen is not only a crucial substrate in the coagulation system but also a classic inflammatory mediator (36). In mediating neuroinflammation, fibrinogen can bind to the CD11b/CD18 integrin receptor to activate the NLRP3 inflammasome in microglial cells, thereby inducing the release of pro-inflammatory cytokines (37–39).

It remains unclear whether intensified blood glucose control should be implemented to manage hyperglycemic AIS patients. One study found that compared to standard blood glucose control, intensified glucose control may further improve hypercoagulability by reducing whole blood tissue factor procoagulant activity, and it was associated with better functional outcomes in AIS patients (40). However, a multicenter, large-sample randomized controlled trial confirmed that intensified blood glucose control did not significantly improve the proportion of AIS patients achieving good functional outcomes at three months compared to standard blood glucose control (41). Furthermore, exenatide therapy was shown to safely reduce the frequency of hyperglycemic events in AIS patients, but it did not alleviate neurological damage (42). Therefore, no treatment plan has yet been established that can effectively improve the prognosis of hyperglycemic AIS patients. Given that chronic hyperglycemia has long-term effects on the coagulation system, the short-term effect of intensified glucose control after AIS onset may be delayed.

High levels of glycosylated hemoglobin may exacerbate thromboinflammation in AIS patients, suggesting that future researchers should focus more on populations with comorbid cerebro-cardiovascular disease and diabetes. This includes conducting in-depth studies on the biological mechanisms by which hyperglycemia induces thromboinflammation, developing cardiovascular event prevention and treatment strategies based on common characteristics within these populations, and verifying the effectiveness of these strategies through multicenter randomized controlled trials. Notably, in this study, less than half of the patients with HbA1c > 6.5% had been previously diagnosed with diabetes. This underdiagnosis of diabetes suggests that regular blood glucose monitoring and control are essential components in the prevention and treatment of stroke in high-risk populations without a prior diabetes history. Furthermore, our subgroup analysis found no significant association between elevated HbA1c levels and hypercoagulability in patients with long-standing diabetes. This suggests that with prolonged duration of diabetes, the coagulation system may become less sensitive to current glycemic control status as reflected by HbA1c. Standard diabetes treatments could modulate the coagulation system, further complicating this relationship.

This study has two limitations. First, while we excluded confirmed cases of active infection, our study could not account for potential subclinical infections that may influence inflammatory markers. Second, our analysis was restricted to admission laboratory tests without subsequent measurements. Future studies should track these measurements over time to understand the temporal progression of HbA1c-associated thromboinflammation.

This study establishes HbA1c as a clinically meaningful biomarker of thromboinflammation in AIS. Although the prognostic utility of HbA1c requires further validation, these findings may directly influence acute-phase treatment strategies, particularly in optimizing antithrombotic therapy. A promising approach would be to combine HbA1c levels with established imaging biomarkers, such as infarct core volume (43), clot burden score (44), and artery occlusion image score (45), during initial assessment. Such integration could enhance early risk stratification and inform personalized antithrombotic interventions.

## 5 Conclusion

An HbA1c level > 6.5% may be an important risk factor for a hypercoagulable state following the onset of AIS, with thromboinflammation potentially playing a key role in the development of this pathological process. Therefore, for AIS patients with HbA1c > 6.5%, close monitoring of coagulation function during hospitalization is essential to prevent the formation of new thrombi.

## Data Availability

The original contributions presented in the study are included in the article/supplementary material. Further inquiries can be directed to the corresponding authors.
